# Dare to Delay? The Impacts of Adolescent Alcohol and Marijuana Use Onset on Cognition, Brain Structure, and Function

**DOI:** 10.3389/fpsyt.2013.00053

**Published:** 2013-07-01

**Authors:** Krista M. Lisdahl, Erika R. Gilbart, Natasha E. Wright, Skyler Shollenbarger

**Affiliations:** ^1^Department of Psychology, University of Wisconsin-Milwaukee, Milwaukee, WI, USA

**Keywords:** adolescence, MRI, alcohol, binge drinking, marijuana, neuropsychology, cognition, age onset

## Abstract

Throughout the world, drug and alcohol use has a clear adolescent onset (Degenhardt et al., 2008). Alcohol continues to be the most popular drug among teens and emerging adults, with almost a third of 12th graders and 40% of college students reporting recent binge drinking (Johnston et al., 2009, 2010), and marijuana (MJ) is the second most popular drug in teens (Johnston et al., 2010). The initiation of drug use is consistent with an overall increase in risk-taking behaviors during adolescence that coincides with significant neurodevelopmental changes in both gray and white matter (Giedd et al., 1996a; Paus et al., 1999; Sowell et al., 1999, 2002, 2004; Gogtay et al., 2004; Barnea-Goraly et al., 2005; Lenroot and Giedd, 2006). Animal studies have suggested that compared to adults, adolescents may be particularly vulnerable to the neurotoxic effects of drugs, especially alcohol and MJ (see Schneider and Koch, 2003; Barron et al., 2005; Monti et al., 2005; Cha et al., 2006; Rubino et al., 2009; Spear, 2010). In this review, we will provide a detailed overview of studies that examined the impact of early adolescent onset of alcohol and MJ use on neurocognition (e.g., Ehrenreich et al., 1999; Wilson et al., 2000; Tapert et al., 2002a; Hartley et al., 2004; Fried et al., 2005; Townshend and Duka, 2005; Medina et al., 2007a; McQueeny et al., 2009; Gruber et al., 2011, 2012; Hanson et al., 2011; Lisdahl and Price, 2012), with a special emphasis on recent prospective longitudinal studies (e.g., White et al., 2011; Hicks et al., 2012; Meier et al., 2012). Finally, we will explore potential clinical and public health implications of these findings.

## Introduction

Throughout the world, drug and alcohol use has a clear adolescent onset (Degenhardt et al., 2008). Alcohol continues to be the most popular drug among teens and young adults, with almost a third of 12th graders and 40% of college students reporting recent binge drinking (four standard alcohol drinks on an occasion in females and five drinks for males; Johnston et al., 2010, 2011). Further, the majority of teens (58%) drinkers also use marijuana (MJ) (Martin et al., 1996), contributing to frequent comorbidity between alcohol and MJ use disorders (Agosti et al., 2002). Indeed, MJ is the second most popular drug and is on the rise in teens, with up to 25% reporting past year use (Johnston et al., 2011). Given this, studies examining the neurocognitive consequences of alcohol and MJ use in youth have gained attention in the scientific literature. This review will present current research regarding the neurocognitive consequences of alcohol, especially binge drinking, and MJ use during the teenage years. Studies utilizing neuropsychological assessment, structural and functional neuroimaging will be reviewed, the impact of teenage drug use onset will be discussed and recommendations for future research will be presented.

Adolescence is a dynamic time marked by increased risk-taking behaviors including substance use (Spear, 2000; Gardener and Steinberg, 2005; Eaton et al., 2006; Casey et al., 2008) that coincide with significant neurodevelopmental changes. Brain regions associated with executive functioning (e.g., problem solving, planning, working memory, and emotional regulation), including the prefrontal cortex (PFC), parietal cortex, and cerebellum, continue to undergo gray matter synaptic pruning into the mid-20s (Giedd et al., 1996a; Sowell et al., 1999, 2002, 2004; Gogtay et al., 2004; Lenroot and Giedd, 2006). White matter volume and integrity increases into the early thirties, yielding improvements in efficient neural conductivity (Giedd et al., 1999; Paus et al., 1999; Barnea-Goraly et al., 2005; Jernigan and Gamst, 2005; Nagel et al., 2006). Scholars have emphasized that it may not be the late maturation of the PFC alone that is responsible for increased risk-taking behavior during adolescence, but rather it is due to differential developmental trajectories of the PFC compared to limbic system. During the teen years, the limbic system develops earlier than the PFC (Giedd et al., 1996b; Galvan et al., 2006; Casey et al., 2008). Indeed, as the PFC undergoes neuronal maturation, greater top-down control of the limbic system results in improved inhibitory control and affective processing as an adolescent becomes an adult (Casey et al., 1997, 2005, 2008; Monk et al., 2003; Liston et al., 2006). It should also be noted that there are gender differences in the timing and rate of neurodevelopment (see Lenroot and Giedd, 2010 for review). More specifically, gray matter volumes peak in executive centers earlier for girls, indicating that females undergo synaptic pruning earlier and there are greater age-related white matter increases in males; overall, this results in relatively larger brain volumes in boys compared to girls (Giedd et al., 1996b; Nagel et al., 2006; Lenroot et al., 2007; Lenroot and Giedd, 2010). This neuromaturation may represent a sensitive period during which exposure to drugs may have a greater impact on neurocognition compared to adult exposure.

## Impact of Adolescent vs. Adult Age of Alcohol Use Onset on Neurocognition

Animal studies have suggested that compared to adults, adolescents may be particularly vulnerable to the neurotoxic effects of early alcohol use onset (AUO) (see Barron et al., 2005; Monti et al., 2005; Spear, 2010 for previous reviews). In humans, addiction specialists have attempted to categorize subtypes of alcohol dependence. One model subdivides alcohol-dependent individuals into Type I and II alcohol-dependent groups (Cloninger, 1987), with Type II alcoholics demonstrating an early AUO (before age 25), earlier treatment attempts, increased novelty seeking, and strong family history of substance-use disorders (SUD; von Knorring et al., 1985; Gilligan et al., 1988; Sullivan et al., 1990). Research examining this typology has revealed that emerging adult AUO (<22–25 years old) is associated with increased childhood behavioral problems, impulsivity, poor decision-making, increased mood disorders, aggressiveness, severity of substance-use problems, more rapid progression from regular drinking to AUD, unique patterns of cerebral blood flow in the PFC, hyperarousal and poor sensorimotor gating, and increased comorbidity with externalizing disorders and ADHD (Varma et al., 1994; Johnson et al., 2000; Demir et al., 2002; Bjork et al., 2004; Dawe et al., 2004; Dom et al., 2006a,b; Pardo et al., 2007; Chen et al., 2011; Lee et al., 2011; Wilens et al., 2011). Specifically, DeWit et al. (2000) reported that the odds of developing lifetime alcohol dependence increase by 14% with each increasing year of AUO.

**Figure 1:**
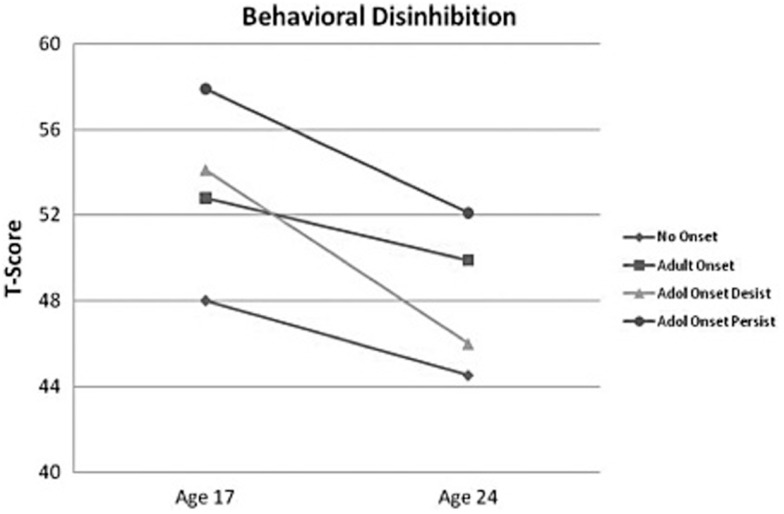
**The figure depicts the mean behavioral disinhibition scores for the alcohol-dependent groups (no onset, *n* = 1211; adult onset, *n* = 545; adolescent onset-desist, *n* = 71; and adolescent onset-persist, *n* = 149) at ages 17 and 24 (scores are in a *T*-score metric; mean = 50, SD = 10)**. Adapted from Hicks et al., 2012; copyright 2011 by*Addiction*.

Several of these symptoms, including impulsivity, poor decision making, externalizing symptoms, aggressiveness, sensation seeking are associated with PFC function, which is continuing to develop during the teenage and emerging adult years (see Kolb et al., 2012; Lenroot and Giedd, 2010 for review). Therefore, it has been hypothesized that PFC dysfunction places individuals at risk for early substance use and early AUO further disrupts PFC development, defining a sensitive period for increased neurocognitive effects in adolescents with AUD. In order to test this model, the Minnesota Twin Family Study examined the impact of premorbid personality and adolescent AUO on personality changes through adolescence into emerging adulthood (Hicks et al., 2010, 2012). These investigations found that behavioral disinhibition prior to AUO significantly predicted age of AUO (no onset, adult onset, adolescent onset who stopped using and adolescent onset with continued symptoms of AUD), with increased disinhibition predicting earlier AUO especially in males (Hicks et al., 2010, 2012). Further, early AUO uniquely predicted lack of maturation in behavioral disinhibition compared to other subgroups (Hicks et al., 2012; see Figure 1). Further, this study found that adolescents who stopped drinking had significant recovery in both behavioral disinhibition and negative emotionality (Hicks et al., 2012), suggesting potential recovery of PFC function with abstinence. Other studies examining the impact of adolescent AUO vs. adult AUO have demonstrated that sensitivity to punishment, disinhibition, and increased likelihood of developing an AUD in teenage AUO (Lyvers et al., 2009, 2011).

## Binge or Heavy Alcohol Use and Neurocognition in Youth

Given the alarming rates of binge drinking in both teenagers and young adults, especially college students (Johnston et al., 2009, 2010), it is important to determine whether binge drinking (defined as four standard alcohol drinks on an occasion in females and five drinks for males), even in the absence of an AUD, is associated with cognition and brain changes. This risky drinking pattern has induced neuronal damage and long-lasting behavioral deficits in adolescent and adult animals (Monti et al., 2005; see Barron et al., 2005; Spear, 2010; Coleman et al., 2011). Still, there have been relatively few human studies to date that specifically examine the effects of intermittent binge drinking in adolescents and emerging adults. Thus far, those studies have reported cognitive deficits associated with binge drinking in otherwise healthy teens and emerging adults, including poorer sustained attention (Hartley et al., 2004), memory (Hartley et al., 2004; Scaife and Duka, 2009; Parada et al., 2011), spatial working memory (Townshend and Duka, 2005; Scaife and Duka, 2009), psychomotor speed (Hartley et al., 2004), working memory (Parada et al., 2012), perseverative responding (Parada et al., 2012), and response inhibition and rule acquisition in females (Townshend and Duka, 2005; Scaife and Duka, 2009), although two studies actually found faster motor responding during a visuospatial task (Townshend and Duka, 2005; Scaife and Duka, 2009). Given the high rates of binge drinking in high school and college students, these results are of great concern and these cognitive problems may be, at least in part, to blame for the lower grades seen in heavy drinking students.

Evidence also suggests underlying structural and functional brain changes associated with binge drinking in adolescents and emerging adults. Using diffusion tensor imaging (DTI), an MRI technique that quantifies white matter integrity, McQueeny et al. (2009) found that teenage binge drinking was associated with significantly reduced white matter quality in several brain regions that connect the brain stem, motor areas, limbic regions, and cortex including the PFC (i.e., the corpus callosum, superior longitudinal fasciculus, corona radiata, internal and external capsules, and commissural, limbic, brainstem, and cortical projection fibers). Greater symptoms of hangover and increased estimated peak BAC estimates were significantly correlated with poorer white matter integrity in white matter tracts connecting the two hemispheres, frontal lobe, and cerebellar tracts.

**Figure 2:**
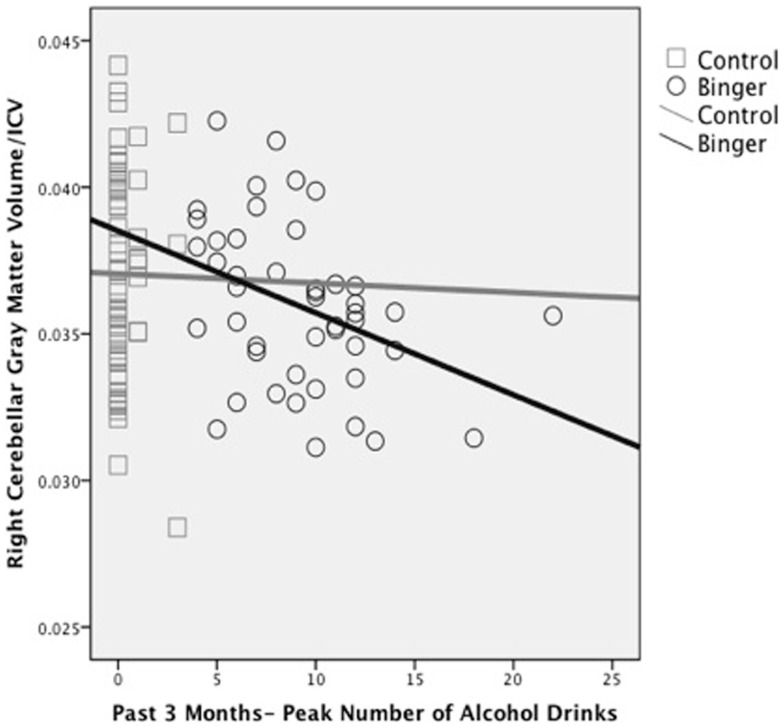
**Reduced right hemisphere cerebellar gray matter volume (corrected for intracranial volume) associated with peak number of alcohol drinks consumed in the past 3 months in binge drinking (*n* = 46) and control (*n* = 60) adolescents (adapted fromLisdahl et al., 2013)**.

Alterations in macro-structure of cortical and subcortical gray matter have also been reported. Although binge drinking was not directly assessed, we (Medina et al., 2010) found that increased overall quantity of alcohol use during the past year was significantly related to smaller cerebellar vermis volumes in substance-using teens. In a follow-up study, our group demonstrated that greater number of drinks per binge in the past 3 months significantly predicted reduced bilateral white and gray matter volumes in the cerebellum in 106 otherwise healthy teens (Lisdahl et al., 2013; see Figure 2). Squeglia et al. (2012) examined cortical thickness in 59 teenagers (ages 16–19) with and without binge-drinking history. Gender significantly moderated the effects of recent binge drinking on PFC and cingulate cortex thickness, with female binge drinkers demonstrating thicker cortices compared to non-drinkers and males demonstrating cortical thinning. In the females, thicker prefrontal cortices were associated with poorer visuospatial, inhibition, and attentional functioning suggesting potential disruption of healthy adolescent PFC pruning in the binge-drinking teens.

Functional changes in brain activation have also been associated with intermittent binge drinking in youth. Event-related potential (ERP) studies have found abnormal signal in anterior and inferior PFC regions to working memory and response inhibition tasks in emerging adults with a history of at least 2 years of intermittent binge drinking (Crego et al., 2010; López-Caneda et al., 2012). Maurage et al. (2009) reported that increases in binge drinking during the first year of college was associated with increasing delays in P1, N2, and P3b latency, areas underlying perceptual, attentional, and executive functioning. This is consistent with Ehlers et al. (2007) who reported smaller P300 amplitudes and latency in adolescents and emerging adults with a binge-drinking history. Research utilizing electroencephalography (EEG) found increased spectral power in delta and fast-beta bands in binge-drinking emerging adults, which is consistent with findings reported in adults with alcohol dependence (Courtney and Polich, 2010).

**Figure 3:**
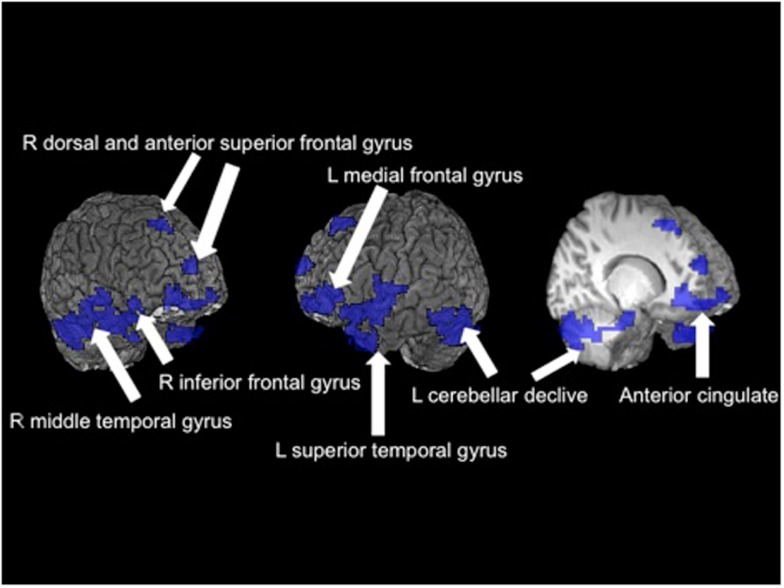
**Significant fMRI clusters predicted by the interaction between gender and binge-drinking status (*N* = 95)**. Areas in blue indicate where female binge drinkers demonstrated significantly reduced BOLD response during the spatial working memory task compared to female controls, while male binge drinkers demonstrated increased BOLD response (adapted fromSqueglia et al., 2011).

In a teenage sample, Schweinsburg et al. (2010a) found that binge drinkers had abnormal brain response during a verbal encoding functional magnetic resonance imaging (fMRI) task. Further, unlike the controls, the binge drinkers failed to engage the hippocampus during novel verbal encoding. In a similar sample of 95 adolescents, Squeglia et al. (2011) reported significant gender differences in binge-drinking effects on a spatial working memory task. Female binge drinkers had blunted activation in frontal, temporal, and cerebellar cortices compared to controls while males demonstrated the opposite pattern (see Figure 3). Other groups have reported blunted amygdala, striatal, and insular activity to emotional cues and decision-making tasks in adolescent binge drinkers compared to social drinkers (Gilman et al., 2012; Xiao et al., 2012).

## Neurocognitive Consequences of Alcohol Use Disorders in Adolescents

Converging lines of evidence suggest that even with substantially shorter periods of exposure, adolescent onset of AUD is associated with neurocognitive deficits. Neuropsychological studies have found that AUD during adolescence and emerging adulthood is associated with poorer verbal memory (Brown et al., 2000; Hanson et al., 2011; Thoma et al., 2011), attention (Tapert and Brown, 1999; Koskinen et al., 2011; Thoma et al., 2011), processing speed (Thoma et al., 2011), visuospatial functioning (Sher et al., 1997; Giancola et al., 1998; Tapert et al., 2002a; Hanson et al., 2011), language (Moss et al., 1994), executive functioning (Hanson et al., 2011; White et al., 2011), and exacerbation of antisocial personality behavior disorder symptoms (Howard et al., 2011). One longitudinal study found that lower levels of impulsive behavior in early adolescence predicted lower rates of AUD in young adulthood; furthermore, they found that past year heavy drinking significantly prospectively predicted additional increases in impulsivity in the following year (White et al., 2011). Withdrawal symptoms seem to be particularly sensitive predictors of cognitive deficits, including poorer visuospatial functioning and memory retrieval (Brown and Tapert, 1999; Brown et al., 2000; Tapert et al., 2002a; Hanson et al., 2011).

Studies utilizing high-resolution MRI have revealed structural abnormalities in teens with AUD, including reduced hippocampal (De Bellis et al., 2000; Nagel et al., 2005; Medina et al., 2007c) and PFC (De Bellis et al., 2005; Medina et al., 2008) volumes, suggesting that adolescent onset of AUD can result in neuronal atrophy, especially in brain regions underlying executive functioning and memory. Using fMRI to assess blood flow changes during cognitive tasks, Tapert et al. (2004) have shown that despite similar behavioral performance on a spatial working memory task, adolescents with AUD have increased brain response in parietal and blunted response in occipital, PFC, and cerebellar regions. Park et al. (2011) found reduced fMRI activation in bilateral frontal and precentral, left superior temporal and parietal cortices, and left cerebellar cortex and increased right uncus activation during a verbal working memory task in teenage males with AUD compared to healthy controls. These results indicate that the adolescent brain may be able to partially compensate for alcohol-induced neuronal insult by relying on other areas to successfully complete the task.

Gender differences in AUD effects have also been reported. Caldwell et al. (2005) found that, after controlling for average BAC, females with AUD demonstrated reduced PFC response compared to gender-matched controls, while the males showed the opposite pattern. Overall, females demonstrated more alcohol-related abnormalities in the PFC compared to males, which was consistent with our structural findings (Medina et al., 2008). Further, young adult women with AUD who underwent a similar fMRI spatial working memory task demonstrated overall blunted brain activation along with poorer behavioral performance (Tapert et al., 2001). In conclusion, emerging adult females with AUD may no longer be able to compensate as effectively as adolescents, demonstrating additional performance decrements with continued alcohol use into early adulthood.

Taken together, these studies suggest that both intermittent binge drinking and the development of AUD can result in significant cognitive, structural, and functional brain changes in both male and female adolescents and emerging adults. Given the fact that approximately 40% of college students engage in binge drinking, this is a major concern. Combined with other alcohol-related consequences (e.g., hangover, poor sleep, emotional stress, legal issues, relationship conflict), these cognitive problems may reduce performance in the classroom. Indeed, studies have found that problematic binge drinking has been predictive of a poorer end-of-semester grade point average (Read et al., 2007).

## Impact of Adolescent vs. Adult Age of Marijuana Use Onset on Neurocognition

**Figure 4:**
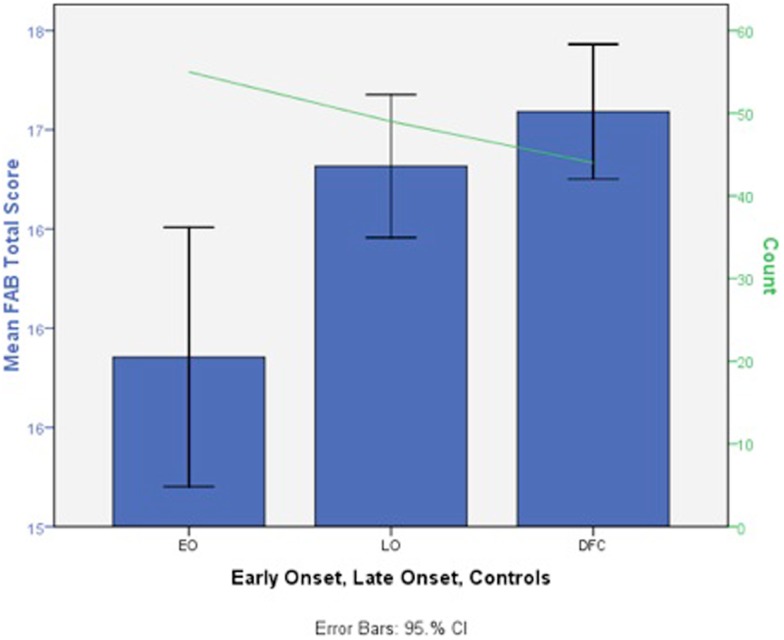
**Deficits in mean total Frontal Assessment Battery (FAB) total score in early adolescent MJ use onset (EO, *n* = 49), late adult onset (LO, *n* = 55), and control groups (DFC, *n* = 44) (scores are in a *T*-score metric; mean = 50, SD = 10 with lower scores indicating impairment; adapted fromFontes et al., 2011)**.

Similar to alcohol findings, preclinical studies have found increased cellular changes associated with THC (delta-9-tetrahydrocannabinol; i.e., one of the major psychoactive compounds in MJ) exposure during adolescence compared to adulthood (e.g., Schneider and Koch, 2003; O’Shea et al., 2004; Cha et al., 2006; Quinn et al., 2008; Rubino et al., 2008). Thus far, human findings suggest that earlier MJ use onset (MUO), typically defined as use starting before 16–18 years old, is associated with more severe cognitive consequences. Converging lines of evidence suggest that regular use of MJ starting before 18 is associated with increased deficits in poorer attention (Ehrenreich et al., 1999), visual search (Huestegge et al., 2002), reduced overall or verbal IQ (Pope et al., 2003; Meier et al., 2012), and executive functioning (Fontes et al., 2011; Solowij et al., 2012). In a thorough study targeting executive functioning, Fontes et al. (2011) compared teenage (*n* = 49) to adult (*n* = 55) MUO matched for IQ, years of daily use, current MJ use, lifetime consumption, and length of abstinence. They found that early onset MJ users had significantly poorer sustained attention, cognitive inhibition, and abstract reasoning (see Figure 4).

Perhaps the most notable study to date on this topic examined the impact of regular MJ use on IQ and neuropsychological functioning in a longitudinal sample of 1,037 individuals followed from birth to age 38 (Meier et al., 2012). After matching for total number of MJ dependence symptoms, the adolescent MUO demonstrated the most robust change in IQ, who as a group demonstrated a drop from childhood “average” to adult “low-average” full-scale IQ. Indeed, the adolescent MUO individuals never achieved their predicted trajectory in IQ, even with sustained abstinence in adulthood.

**Figure 5:**
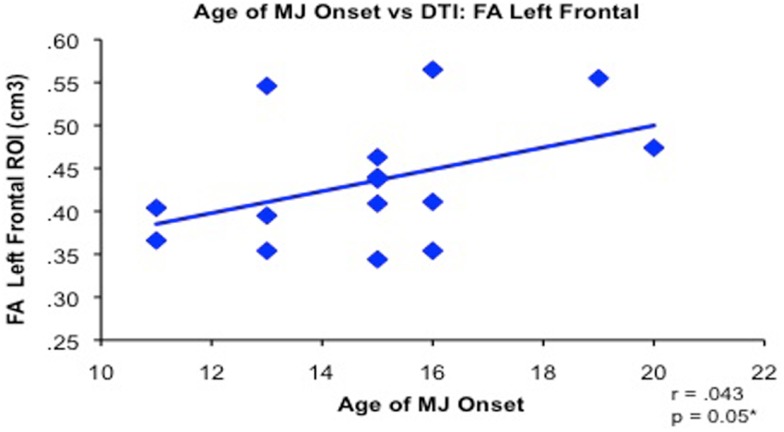
**Bivariate relationship between younger age of regular marijuana (MJ) use onset (range 11–20 years of age) and decreased white matter integrity (reduced FA measured by diffusion tensor imaging) in 15 MJ users in the left frontal region of interest (adapted fromGruber et al., 2011)**.

**Figure 6:**
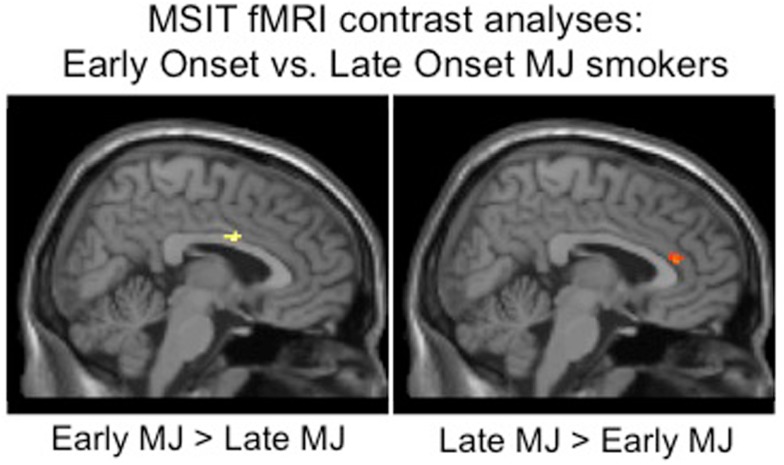
**Whole brain inhibitory processing results demonstrating significant differences between adolescent early onset (*n* = 9) and late adult onset (*n* = 14) MJ users, early onset MJ users demonstrated increased middle right cingulum and decreased anterior cingulate BOLD response to an inhibitory processing (multisource interference task, MSIT) fMRI task (adapted fromGruber et al., 2012)**.

Increased structural and functional brain changes associated with adolescent MUO have also been reported. In one of the earliest studies, Wilson et al. (2000) found reduced overall cortical gray matter and increased white matter volumes in participants with adolescent MUO compared to later onset of use. Lopez-Larson et al. (2011) found significant correlations between earlier MUO and decreased right superior PFC cortical thickness in 18 current MJ users. Adolescent onset MJ use has also been linked with increased PFC white matter diffusivity and increased impulsivity compared to later onset in a sample of well-matched MJ users (Gruber et al., 2011; see Figure 5). Functional MRI studies have reported abnormal brain activation abnormalities in early vs. late MUO in PFC and parietal regions (Becker et al., 2010a; Jager et al., 2010; Gruber et al., 2012), although one study did not report age of onset effects on a verbal encoding task (Becker et al., 2010b). [See Figure 6 to examine PFC activation differences between adolescent and adult MUO groups on an inhibitory control fMRI task (Gruber et al., 2012)].

In summary, the brain appears to be particularly vulnerable to adolescent MJ exposure. The PFC continues to mature into early adulthood and may be particularly sensitive to adolescent MJ exposure, as early MUO samples have demonstrated executive dysfunction (Fontes et al., 2011; Gruber et al., 2011; Solowij et al., 2012), structural damage (Churchwell et al., 2010; Gruber et al., 2011; Lopez-Larson et al., 2011), and abnormal brain activation (Jager et al., 2010; Gruber et al., 2012) in the PFC.

## Heavy Marijuana Use and Neurocognition in Adolescents and Emerging Adults

Consistent with the age of onset data, converging lines of evidence is building to suggest that chronic MJ during the teenage years is associated with neurocognitive deficits. For example, in a longitudinal study following adolescents with SUD over time, Tapert et al. (2002b) found that greater cumulative MJ use over an 8-year follow-up period was associated with poorer attention functioning. Tait et al. (2011) found that after controlling for potentially confounding variables, continued MJ use over an 8-year period was associated with decrements in verbal memory. Other studies conducted in adolescents with minimal psychiatric comorbidities have suggested cognitive deficits associated with regular adolescent MJ use, including processing speed (Fried et al., 2005; Medina et al., 2007a; Lisdahl and Price, 2012), complex attention (Tapert et al., 2002a; Harvey et al., 2007; Medina et al., 2007a; Hanson et al., 2010b; Mathias et al., 2011; Lisdahl and Price, 2012), memory (Schwartz et al., 1989; Fried et al., 2005; Harvey et al., 2007; Medina et al., 2007a; McHale and Hunt, 2008; Hanson et al., 2010b; Solowij et al., 2011; Tait et al., 2011; Thoma et al., 2011), executive functioning, especially cognitive disinhibition (Harvey et al., 2007; Medina et al., 2007a; McHale and Hunt, 2008; Hanson et al., 2010b; Mathias et al., 2011; Gonzalez et al., 2012; Grant et al., 2012; Lisdahl and Price, 2012; Schuster et al., 2012; Solowij et al., 2012), and risky sexual behavior (Schuster et al., 2012).

**Figure 7:**
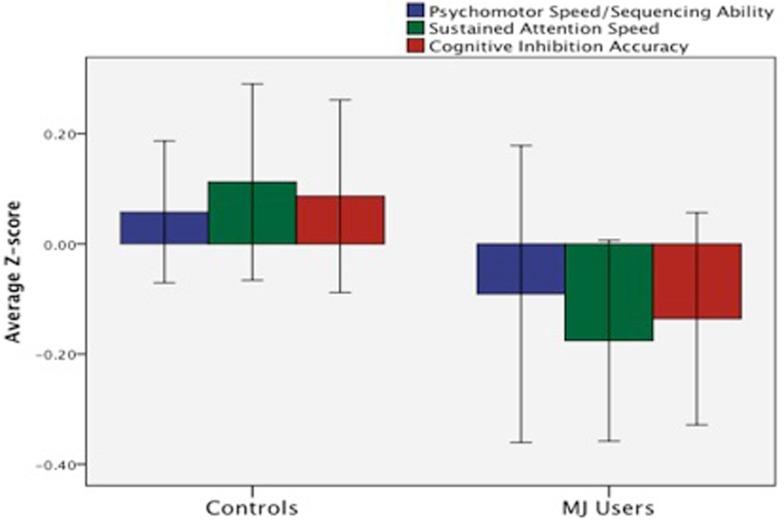
**Deficits in mean *z*-score psychomotor speed, sustained attention, and cognitive inhibition were observed in emerging MJ (*n* = 23) users compared to normal controls (*n* = 35) following a minimum of 1 week of abstinence (adapted fromLisdahl and Price, 2012)**.

We (Medina et al., 2007a) compared neuropsychological functioning in a sample of demographically matched healthy controls and MJ-using adolescents without comorbid psychiatric disorders who underwent 28 days of monitored abstinence. After controlling for alcohol use, adolescent MJ users demonstrated deficits in complex attention, verbal story learning, sequencing ability, and slower psychomotor speed compared to controls (Medina et al., 2007a). In a follow-up study that included 59 teens and emerging adult MJ users and controls, we found a similar pattern of cognitive deficits in the MJ users who demonstrated poorer complex attention, slower psychomotor speed, and reduced inhibitory control (Lisdahl and Price, 2012; see Figure 7).

Increasingly, studies utilizing neuroimaging methods to assess brain structure have reported consequences of chronic MJ use in adolescents. Our group has examined brain volumes in a sub-sample of adolescent MJ users without comorbid psychiatric, developmental, or neurologic conditions (ages 16–19) and healthy controls. Thus far, we found that adolescent MJ users (who also had heavy alcohol use) did not significantly differ from healthy controls in their hippocampal volumes, although correlations between hippocampal volumes and verbal memory were abnormal compared to the controls (Medina et al., 2007c). In 16 MJ users and 16 healthy controls without comorbid psychiatric disorders we found marginal MJ group-by-gender interactions in predicting PFC volume; female MJ users demonstrated comparatively larger volumes, while male users had smaller volumes compared to same-gender controls (Medina et al., 2009). MJ group status and total PFC volume interacted in predicting executive functioning; among the MJ users (especially the girls), larger PFC volumes were associated with poorer executive functioning, while the opposite pattern was seen among the controls, suggesting that larger PFC volumes in the MJ users was detrimental. More recently, increased posterior inferior cerebellar vermis volumes in adolescent MJ users and increased left amygdala volumes in female MJ users were observed compared to controls, suggesting disruption in affective processing circuitry (Jarvis et al., 2008; Medina et al., 2010; McQueeny et al., 2011).

Recently other groups have reported decreased cortical thickness in right caudal middle frontal, bilateral insula, and bilateral superior frontal cortices and increased cortical thickness in lingual, temporal, inferior parietal, and paracentral regions (Lopez-Larson et al., 2011), decreased right medial orbitofrontal cortex volume (Churchwell et al., 2010), and reduced bilateral hippocampal volumes (Ashtari et al., 2011) in adolescent MJ users without comorbid psychiatric conditions compared to healthy controls. The above structural alterations were associated with increased executive dysfunction (Medina et al., 2009, 2010; Churchwell et al., 2010), mood symptoms (McQueeny et al., 2011), and verbal memory deficits (Ashtari et al., 2011). Adolescent MJ users have also demonstrated reduced cerebral blood flow in temporal, insular, and PFC regions after 4 weeks of monitored abstinence, which may also underlie observed cognitive deficits (Jacobus et al., 2012).

Micro-structural and neurochemical abnormalities have also been reported in otherwise healthy adolescent MJ users. Recent use of magnetic resonance spectroscopy (MRS) has revealed neurochemical alterations in adolescent MJ users, including reduced anterior cingulate glutamate, *N*-acetyl aspartate, creatine, and *myo*-inositol (Prescot et al., 2011), lower global *myo*-inositol/creatine ratios in subcortical gray matter structures, and reduced *myo*-inositol in white matter (Silveri et al., 2011) suggesting an early neurochemical response to neuronal toxicity and disruption of microglia activity.

Subtle white matter abnormalities have also been observed in adolescent and emerging adult MJ users. Our group found that increased depressive symptoms in MJ users was associated with smaller global white matter volume (Medina et al., 2007b), suggesting that MJ use during adolescence may disrupt white matter connections between areas involved in mood regulation. Using DTI, Bava et al. (2009) found that MJ users had significantly poorer white matter integrity, measured by lower fractional anisotropy (FA) in 10 brain regions, especially in regions underlying executive functioning and working memory. Increased FA was also seen in regions underlying vision, suggesting possible over-recruitment of these brain regions in adolescent MJ users compared to controls. With one exception (DeLisi et al., 2006), these results are consistent with other studies that have demonstrated reduced white matter integrity in adolescent and young adult MJ users who initiated use during adolescence (Arnone et al., 2008; Ashtari et al., 2009; Gruber et al., 2011).

There is also converging evidence of inefficient brain activation patterns in adolescent and emerging adult MJ users compared to healthy controls. Studies utilizing fMRI and PET with adolescents have found abnormal PFC, limbic, parietal, and cerebellar activation patterns in MJ users in response to finger tapping (Lopez-Larson et al., 2012), attentional control (Abdullaev et al., 2010), verbal working memory (Jacobsen et al., 2007; Jager et al., 2010), verbal encoding (Becker et al., 2010b), spatial working memory (Schweinsburg et al., 2008, 2010b; Smith et al., 2010), cognitive inhibition (Tapert et al., 2007), and monetary decision-making (Vaidya et al., 2012) tasks. For example, Jager et al. (2010) reported that MJ-using teenage boys (ages 13–19) demonstrated excessive activation in executive (PFC) regions during a verbal working memory task, especially during initial encoding, compared to non-using healthy controls. Consistent with this finding, our laboratory (Tapert et al., 2007) found that after controlling for alcohol use, MJ users demonstrated increased executive (right dorsolateral PFC, bilateral medial frontal), working memory (parietal), and visual (occipital) activation during inhibitory “no-go” trials (i.e., tests of impulse control), compared to normal controls, even though they had marginally poorer performance. Further, teen MJ users with lighter use histories demonstrated the greatest brain activation to both the cognitive inhibition and spatial working memory tasks (Tapert et al., 2007; Schweinsburg et al., 2008), while teens with more intense use histories (earlier onset, longer duration, increased lifetime use) had lower activation than controls. A recent functional connectivity study found increased connectivity between PFC and occipitoparietal regions in adolescent MJ users as cognitive control demands increased (Harding et al., 2012). These findings suggests that during *initial* MJ exposure the brain may successfully compensate by recruiting additional neuronal resources, although this compensation may falter with more problematic and increased MJ use patterns.

Taken together, the above studies suggest that regular MJ use during adolescence may lead to structural changes such as abnormal gray matter pruning patterns and reduced white matter myelination. These changes have been associated with poor neuronal efficiency and poorer cognitive functioning, especially psychomotor speed, executive functioning, emotional control, and learning and memory, even after a month of monitored abstinence. Given the high rates of MJ use in teens and emerging adults, this may mean a large proportion of youth are experiencing cognitive difficulties that may negatively impact their performance. Indeed, we have found increased school difficulty and reduced grades in MJ-using teens (Medina et al., 2007a) (Table 1).

**Table 1:** **Human studies reporting neurocognitive effects of regular alcohol and marijuana exposure in adolescents and emerging adults (organized by cognitive, structural, or functional consequences and clustered according to functional outcomes)**.

Alcohol use disorder studies	Teenage onset worse?	Cognitive deficits	Brain structure abnormalities	Brain function abnormalities
Hicks et al. (2012)	Yes	↑ behavioral disinhibition		
Lyvers et al. (2009)	Yes	↑ reward sensitivity; disinhibition		
Lyvers et al. (2011)	Yes	↑ reward sensitivity; disinhibition		
Brown et al. (2000)		↓ verbal memory		
Hanson et al. (2011)		↓ verbal memory		
Thoma et al. (2011)		↓ processing speed		
Koskinen et al. (2011)		↓ attention		
Tapert and Brown (1999)		↓ attention		
Giancola et al. (1998)		↓ visuospatial ability		
Sher et al. (1997)		↓ visuospatial ability		
Tapert et al. (2002a)		↓ visuospatial ability		
Moss et al. (1994)		↓ language		
White et al. (2011)		↓ executive functioning, inhibition		
Howard et al. (2011)		↑ antisocial personality disorder symptoms		
De Bellis et al. (2000)			↓ HC volume	
Nagel et al. (2005)			↓ left HC volume	
Medina et al. (2007a)			↓ left HC volume	
Medina et al. (2010)			↓ cerebellar vermis GM volume	
De Bellis et al. (2005)			↓ PFC volume	
Medina et al. (2008)			↓ PFC volume	
Caldwell et al. (2005)				Females: ↓ superior frontal, temporal, cingulate, fusiform BOLD response during SWM task; Males opposite pattern.
Park et al. (2011)				↓ PFC, temporal, parietal, cerebellar, ↑uncus fMRI BOLD during VWM task in males
Tapert et al. (2004)				↓ PFC, occipital, cerebellar, ↑parietal fMRI BOLD during SWM task
Tapert et al. (2001)				↓ PFC, parietal fMRI BOLD during SWM task in females

**Binge-drinking studies**	**Teenage onset worse?**	**Cognitive findings**	**Brain structure findings**	**Brain function findings**

Hartley et al. (2004)		↓ sustained attention, memory, psychomotor speed		
Parada et al. (2011)		↓ verbal memory, working memory, perseverative responding		
Scaife and Duka (2009)		↓ verbal memory, SWM, cognitive inhibition		
Townshend and Duka (2005)		↓ SWM, cognitive inhibition, rule acquisition		
Lisdahl et al. (2013)			↓ L/R cerebellar GM and WM volumes	
McQueeny et al. (2009)			↓ white matter integrity DTI (CC, superior longitudinal fasciculus, corona radiate, internal/external capsules)	
Squeglia et al. (2012)			Females: ↑ PFC/cingulate thickness; Males: ↓PFC/cingulate thickness	
Courtney and Polich (2010)				↑EEG spectral power in delta and fast beta bands
Crego et al. (2010)				↓ERP in anterior/inferior PFC
Ehlers et al. (2007)				↓P300 ERP amplitude
López-Caneda et al. (2012)				↑go-P3 ERP in right inferior PFC
Maurage et al. (2009)				↓P1, N2, P3b ERP latency
Gilman et al. (2012)				↓NAcc, amygdala fMRI BOLD during emotional cues task after consuming alcohol
Schweinsburg et al. (2010a)				↓HC fMRI BOLD during verbal encoding task
Squeglia et al. (2011)				Females: ↓PFC, temporal, and cerebellar BOLD during SWM fMRI task. Males: opposite pattern.
Xiao et al. (2012)				↑amygdala, insula fMRI BOLD during IGT task

**Marijuana studies**	**Teenage onset worse?**	**Cognitive findings**	**Brain structure findings**	**Brain function findings**

Meier et al. (2012)	Yes	↓ IQ		
Pope et al. (2003)	Yes	↓ IQ		
Ehrenreich et al. (1999)	Yes	↓ attention		
Huestegge et al. (2002)	Yes	↓ visual search		
Fontes et al. (2011)	Yes	↓ executive functioning		
Solowij et al. (2012)	Yes	↓ executive functioning		
Churchwell et al. (2010)	Yes		↓ PFC volume	
Gruber et al. (2011)	Yes	↑ impulsivity	↓ WM integrity in PFC	
Lopez-Larson et al. (2011)	Yes		↓ superior PFC thickness	
Wilson et al. (2000)	Yes		↓ total GM; ↑total WM	
Becker et al. (2010a)	Yes			↑left superior PFC fMRI BOLD during working memory task in early onset
Becker et al. (2010b)	No			↑left parahippocampal gyrus, fMRI BOLD during learning task in all MJ users
Gruber et al. (2012)	Yes			↓anterior cingulate fMRI BOLD during inhibition task in early onset
Jager et al. (2010)	Yes			↑PFC fMRI BOLD during novel stimuli presentation in working memory task in early onset
Fried et al. (2005)		↓ processing speed verbal memory		
Hanson et al. (2010b)		↓ complex attention, verbal memory		
Harvey et al. (2007)		↓ complex attention, verbal memory; executive functioning		
Lisdahl and Price (2012)		↓ complex attention processing speed, sequencing ability, cognitive inhibition		
Medina et al. (2007a)		↓ complex attention processing speed, verbal memory, sequencing ability		
Mathias et al. (2011)		↓ complex attention, executive functioning		
Tapert et al. (2002a)		↓ complex attention		
McHale and Hunt (2008)		↓ verbal memory, executive functioning		
Schwartz et al. (1989)		↓ verbal memory		
Solowij et al. (2011)		↓ verbal memory; executive functioning		
Tait et al. (2011)		↓ verbal memory		
Thoma et al. (2011)		↓ verbal memory		
Gonzalez et al. (2012)		↓ executive functioning		
Grant et al. (2012)		↓ executive functioning		
Schuster et al. (2012)		↓ executive functioning; ↑risky sexual behavior		
McQueeny et al. (2011)		↑ depressive symptoms	Females: ↑left amygdala	
Medina et al. (2007b)		↑ depressive symptoms	↓global WM	
Jarvis et al. (2008)			↑amygdala volume	
Ashtari et al. (2011)		↓ verbal memory	↓HC volume	
Medina et al. (2007b)			↑left HC volume	
Churchwell et al. (2010)		↓ executive functioning	↓right medial orbitofrontal cortex volume	
Lopez-Larson et al. (2011)			↓right caudal, middle frontal, inula, superior frontal thickness; ↑lingual, temporal, inferior parietal, paracentral thickness	
Medina et al. (2010)		↓ executive functioning	↑inferior cerebellar vermis volume	
Medina et al. (2009)		↓ executive functioning	Females: ↑inferior PFC volume	
Arnone et al. (2008)			↓WM integrity (corpus collosum)	
Ashtari et al. (2009)			↓WM integrity (arcuate fasciculus)	
Bava et al. (2009)			↓white matter integrity in 10 regions (especially PFC, parietal cortex); ↑WM integrity in occipital cortex	
DeLisi et al. (2006)			No WM differences detected	
Prescot et al. (2011)			↓ACC glutamate, N-acetyl aspartate, creatine, *myo*-inositol	
Silveri et al. (2011)			↓subcortical GM *myo*-inositol/creatine; WM *myo*-inositol	
Abdullaev et al. (2010)				↑PFC fMRI BOLD during attentional control task
Harding et al. (2012)				↑ PFC and occipitoparietal connectivity as task demands increase
Jacobsen et al. (2007)				↓ PFC, parietal connectivity during verbal working memory task while undergoing nicotine withdrawal
Jacobus et al. (2012)				↓ cerebral blood flow in temporal lobe, insula, and PFC
Jager et al. (2010)				↑ PFC fMRI BOLD during verbal encoding task in males
Lopez-Larson et al. (2012)				↓ cingulate gyrus, cerebellar fMRI BOLD during finger tapping task
Schweinsburg et al. (2008)				↓ PFC, occipital, ↑parietal fMRI BOLD during SWM task
Schweinsburg et al. (2010b)				↑ PFC, insula, ↓precentral fMRI BOLD during SWM task in recent MJ users vs. abstinent users
Smith et al. (2010)				↑ inferior, middle PFC fMRI BOLD during SWM task
Tapert et al. (2007)				↑ PFC, parietal, occipital fMRI BOLD during inhibitory processing task
Vaidya et al. (2012)				↑ ventral medial PFC, cerebellar PET rCBF during IGT task

*Teenage onset worse? = “yes” – analysis revealed that teenage age of onset (<16, 17, or 18 years of age) was associated with significantly poorer neurocognitive outcome; if “no” – onset was not associated with outcome; if left blank – age of onset analysis was not conducted in this study. GM, gray matter; WM, white matter; PFC, prefrontal cortex; HC, hippocampus; SWM, spatial working memory; VWM, verbal working memory; IGT, Iowa Gambling task*.

## Potential Limitations of the Existing Literature

It is important to note some limitations of the above research. Although several of the above studies did control for family history of SUD and excluded subjects with Axis I comorbid psychiatric disorders, it is still difficult to determine whether the brain and cognitive abnormalities may have predated the onset of adolescent drug use. Risk factors associated with early drug experimentation (such as poor cognitive inhibition, attention problems, conduct disorder, and family history of SUD) are themselves related to subtle cognitive and brain abnormalities (Aronowitz et al., 1994; Tapert and Brown, 2000; Tapert et al., 2002a; Nigg et al., 2004; Schweinsburg et al., 2004; Hill et al., 2007a,b; Spadoni et al., 2008; Ridenour et al., 2009; Hanson et al., 2010a) and at least some evidence exists suggesting preexisting brain abnormalities predate and predict the onset of substance use (e.g., Cheetham et al., 2012). It is notable, however, that prospective longitudinal studies have provided evidence for additional cognitive and brain abnormalities following the onset of regular alcohol or MJ use that are above and beyond premorbid differences in personality, cognition, and brain structure (Maurage et al., 2009; Hicks et al., 2012; White et al., 2011; Meier et al., 2012). Still, additional longitudinal research in teenagers prior to alcohol and MJ exposure, especially in at-risk comorbid samples, is needed to explore the influence of early drug use on adolescent neurodevelopment.

## Recovery of Function with Abstinence? A Message of Hope

There is even less research available to help determine whether sustained abstinence from alcohol and MJ results in recovery of cognitive functions, although findings to date are hopeful. For example, Hanson et al. (2011) reported that having greater days of abstinence from alcohol and drugs at a 10-year follow-up was associated with improved executive functioning, even controlling for baseline executive functioning and education. In our binge-drinking sample, increased abstinence was associated with larger bilateral cerebellar volumes (Lisdahl et al., 2013). In adolescent MJ users, short-term memory impairments mildly recovered following 3–6 weeks of MJ abstinence (Schwartz et al., 1989; Hanson et al., 2010b), although another study found that adolescent MJ users who abstained for a minimum of 3 months did not demonstrate any cognitive deficits compared to controls (Fried et al., 2005) and in one prospective longitudinal study individuals who began using MJ early never returned to their predicted IQ trajectory even with sustained abstinence in adulthood (Meier et al., 2012). Few fMRI studies have examined recovery of function; in a cross-sectional study, recent MJ users demonstrated increased activation in brain regions underlying executive control and attention, such as the insula and PFC, compared to abstinent ex-users (Schweinsburg et al., 2010b). This preliminary evidence suggests that the inefficient brain response seen in teenage MJ users may begin to normalize after several weeks of abstinence. In sum, these results suggest there may be subtle recovery of cognitive functioning with increasing lengths of abstinence from MJ and alcohol. Additional research is necessary to examine whether complete recovery of neurocognitive functioning occurs in adolescents with sustained abstinence, or if their neurocognitive trajectory is subtly altered into adulthood. Still, these preliminary findings can be utilized to help increase motivation for abstinence in alcohol and MJ-using youth, as it is expected that with continued abstinence they will experience at least minimal improvements in attention, verbal memory, and neuronal processing speed.

## Conclusion and Recommendations

### Increase psychoeducation, screening, and personalized feedback

Alarming numbers of adolescents and emerging adults regularly binge drink and use MJ (Johnston et al., 2009, 2010). Animal and human research suggests that adolescence may be a vulnerable period for drug exposure due to critical neurodevelopmental processes that peak during this period. Indeed, adolescents and emerging adults who initiate binge drinking or use MJ regularly tend to show inferior cognitive skills compared to teens that abstain or use lightly or Compared to individuals who begin substance use in adulthood. This review paper outlined several studies that suggest binge drinking, AUD, and chronic MJ use during the teenage and early adult years results in gray and white matter micro- and macro-structural abnormalities that are oftentimes correlated with cognitive deficits. Evidence is also mounting that heavy teenage alcohol and MJ use may disrupt brain function, leading to inefficient neuronal activation early on and diminished activation with continued heavy use into emerging adulthood. Additional research is needed to examine the impact of these neurocognitive deficits on treatment outcomes in order to individualize treatment and prevention campaigns (e.g., Feldstein Ewing et al., 2012).

These findings have significant clinical impact as even subtle brain abnormalities and cognitive problems in teens and young adults may lead to important psychosocial consequences. Combined negative impacts of drug and alcohol-related consequences (such as hangovers or emotional stress), sleep deprivation caused by drug use (Cohen-Zion et al., 2009), and acute effects of being intoxicated at school may lead to even more pronounced cognitive problems in *current* alcohol and MJ-using college students. Youth may miss information presented in class or on the job due to poorer processing speed, initial learning, complex attention, and working memory. Indeed, researchers have found that substance-induced cognitive disadvantage may lead to lower than expected school performance, increased school problems, risky decision-making, and poorer emotional regulation (Lynskey and Hall, 2000; Medina et al., 2007a; Kloos et al., 2009).

It is critical to disseminate these findings to high school and college students, young military enlistees, therapists, teachers, child psychiatrists, pediatricians, and parents to help minimize regular alcohol and MJ consumption in youth. Fortunately, high-quality psychoeducation materials regarding the effects of alcohol and drugs on the brain, including pamphlets designed for teens and young adults, are available at no cost through the National Institute on Drug Abuse^1^, the National Institute on Alcohol Abuse and Alcoholism^2^, teen-centered sites like the www.thecoolspot.gov and www.drugfreeamerica.org, and university websites such as Teen Safe^3^, which has an excellent parent resource center. Still, we may improve outcomes by providing more personalized feedback about drugs and alcohol health effects (see Larimer and Cronce, 2007). To date, however, no systematic individualized feedback programs have integrated information regarding the effects of drugs on neurocognition. At this time, more global feedback focused on group, or normative, performance results could be integrated. For example, adolescents who engage in heavy drinking could be told that, “Teens who drank more than nine alcohol drinks in one occasion had 1.8 cubic centimeters less cerebellar brain volume than teens who drank three or fewer drinks when drinking, on average. The cerebellum is important for coordination and thinking skills” (Lisdahl et al., 2013). Youth who engage in weekly MJ use could be told “even with similar verbal intelligence and reading ability, MJ users scored more than half a standard deviation lower on an executive functioning task, achieved a half-point lower GPA, and were more likely to demonstrate behavioral problems in school (26 vs. 0%) compared to peers who did not regularly use MJ” (Medina et al., 2007a). This normative feedback could be developed further and disseminated more globally by services aimed at health education and drug prevention in youth. One potential opportunity is to integrate this information more thoroughly into existing computerized programs such as CRAFFT screening tool (Knight et al., 2002), which asks six questions and reveals a teen’s risk for problematic, abusive, or dependent use patterns^4^. After retrieving your score, the computerized program provides potential impact of your use on health, including brain function. After taking the screening tool, physicians and therapists could then utilize brief motivational interviewing to help educate youth further about the negative effects of alcohol and MJ use on the brain. Taken further, therapists could order neuropsychological testing and give truly individualized feedback regarding the student’s cognitive functioning.

### Develop interventions to improve neurocognition: Exercise?

Treatments that may reverse substance-induced neurocognitive damage in youth are needed. Some potential candidates include cognitive rehabilitation (see Macher and Earleywine, 2012) or exercise. In animals, physical activity has been linked to decreased inflammatory response and oxidative stress at moderate levels (Radak et al., 2007; Sim et al., 2008; Sakurai et al., 2009), increased c-FOS expression (Sim et al., 2008), and improved catecholaminergic (dopamine, norepinephrine, and epinephrine) function in brain regions including the PFC (Heyes et al., 1985; Elam et al., 1987; Chaouloff, 1989; Dunn and Dishman, 1991; Dunn et al., 1996; Waters et al., 2005). Several human studies have concluded that activity and cardiorespiratory fitness have positive effects on brain health and neuronal plasticity, although the vast majority of the studies have been conducted in older adults (Brisswalter et al., 2002; Cotman and Berchtold, 2002; Colcombe and Kramer, 2003; Colcombe et al., 2004, 2006; Heyn et al., 2004; Kramer and Erickson, 2007; Boecker et al., 2008; Hillman et al., 2008; Ma, 2008; Ploughman, 2008; Coelho et al., 2013). Given ongoing neurodevelopment and fewer comorbid problems like vascular disease in youth, these findings may not directly generalize to teens.

Although research has shown that physical activity is associated with improved mood, decreased drug use, and increased grade point in adolescents (Winnail et al., 1995; Field et al., 2001; Audrain-McGovern et al., 2006), very few studies have directly examined the neurocognitive benefits of physical activity in adolescents. In meta-analyses (Etnier et al., 1997; Sibley and Etnier, 2003), low to large (0.24–0.77) effect sizes for the impact of activity on perceptual skills, academic achievement, and verbal tests in adolescents have been reported; however, higher-order executive functioning or brain structure were not measured. Research examining the impact of acute effects of exercise or improved fitness in healthy emerging adults have found superior executive control (Dustman et al., 1990; Hillman et al., 2003; Themanson and Hillman, 2006; Themanson et al., 2006; Ferris et al., 2007), increased cerebral blood flow (Pereira et al., 2007; Timinkul et al., 2008), and improved white matter integrity (Marks et al., 2007). In sum, there is at least preliminary evidence that increased physical activity is associated improved neurocognitive functioning, especially executive functioning, in otherwise healthy young adults without cerebrovascular disease. Perhaps most promising, recent research has suggested that exercise interventions may reverse neuronal damage in binge drinking adolescent animals (Helfer et al., 2009) and brief interventions to increase exercise may help reduce drug use and increase physical activity in adolescents (Werch et al., 2005). Additional research is needed to examine how physical activity impacts neurocognition in adolescent drug users, but there is optimism that this is an ideal time to intervene. Indeed, physical activity during the this sensitive stage of ongoing neurodevelopment (ages 15–25) has been associated with superior information processing in elderly men, after controlling for their current level of activity (Dik et al., 2003). Therefore, there is an opportunity to intervene early during the school years to reduce drug use, reverse neurocognitive damage, and perhaps instill lifelong exercise habits that may actually improve aging.

### Summary: Delay the onset

Adolescence has been named the “gateway to adult health outcomes” (Raphael, 2013) and presents a golden opportunity for public policy intervention to significantly improve health outcomes that last throughout adulthood. However, this sensitive period is also associated with the onset of binge drinking and MJ use, which negatively impacts cognition, brain structure, and function in otherwise healthy teens and young adults. Early age of onset (before age 18) has been linked with the greatest neurocognitive deficits. Therefore, general psychoeducation coupled with personalized feedback regarding effects of chronic drug use on thinking abilities and brain health need to be integrated into current prevention, screening, and treatment programs. Interventions geared toward lowering alcohol and drug exposure in teens and young adults that have shown evidence of efficacy need to be implemented more aggressively in schools and college campuses to not only reduce symptoms of drug abuse and dependence, but *delay the onset of regular use* from early teen years to early adult years in order to prevent long-term neuronal damage and ensure optimal brain health and cognitive functioning in youth.

## Conflict of Interest Statement

The authors declare that the research was conducted in the absence of any commercial or financial relationships that could be construed as a potential conflict of interest.

## Footnotes

^1^www.nida.nih.gov

^2^www.niaaa.nih.gov

^3^www.Teen-Safe.org

^4^http://www.ceasar.org/teens/test.php
